# Digital Image Analysis Tools Developed by the Indiana O’Brien Center

**DOI:** 10.3389/fphys.2021.812170

**Published:** 2021-12-16

**Authors:** Kenneth W. Dunn

**Affiliations:** Division of Nephrology, Indiana University School of Medicine, Indianapolis, IN, United States

**Keywords:** image analysis, volume rendering, segmentation, tissue cytometry, intravital microscopy, image registration

## Abstract

The scale and complexity of images collected in biological microscopy have grown enormously over the past 30 years. The development and commercialization of multiphoton microscopy has promoted a renaissance of intravital microscopy, providing a window into cell biology *in vivo*. New methods of optical sectioning and tissue clearing now enable biologists to characterize entire organs at subcellular resolution. New methods of multiplexed imaging support simultaneous localization of forty or more probes at a time. Exploiting these exciting new techniques has increasingly required biomedical researchers to master procedures of image analysis that were once the specialized province of imaging experts. A primary goal of the Indiana O’Brien Center has been to develop robust and accessible image analysis tools for biomedical researchers. Here we describe biomedical image analysis software developed by the Indiana O’Brien Center over the past 25 years.

## Introduction

Over the past 200 years, biological microscopy has evolved from a largely descriptive technique, documented with pictures and verbal descriptions, into a legitimately quantitative research approach. This evolution was fueled by the widespread deployment of digital detectors in the 1980s and digital computers in the 1990s. As biological microscopy became “digital,” biologists increasingly found themselves having to train themselves in methods of digital image analysis in order to visualize and analyze their imaging studies. The past 20 years have witnessed an extraordinary explosion in the development of methods of biological microscopy, extending its scope, scale, complexity and resolution. Realizing the vast potential of these techniques has required that biomedical researchers master increasingly challenging methods of image and data analysis, methods that are generally well outside the realm of their training. Over the course of the Indiana O’Brien Center’s existence [see review in Dunn et al. (2021)], we have encountered multiple cases where necessary software tools either do not exist or require an inordinately high level of expertise. A primary goal of the Indiana O’Brien Center has been to develop robust image analysis tools that are accessible to biomedical researchers lacking specialized image analysis experience. Examples of image analysis software developed by the Center are listed in Table 1, and described in detail below.

**TABLE 1:** Software developed by the Indiana O’Brien Center.

Software	Application	References	Availability
Voxx	3D volume rendering for personal computers	Clendenon et al., 2002. *Am J Physiol Cell Physiol*. 282:C213-218	http://web.medicine.iupui.edu/ICBM/software
IMART	Motion correction for time-series and 3D intravital microscopy images	Dunn et al., 2014. *Intravital*. 3:e28210 Lorenz et al., 2012. *J Microsc*. 245:148-160	http://web.medicine.iupui.edu/ICBM/software
STAFF	Near-continuous measurement of microvascular velocity in 2D networks	Clendenon et al., 2019b. *Microvasc Res*. 123:7-13 Clendenon et al., 2019a. *J Vis Exp*	https://github.com/icbm-iupui/STAFF
VTEA	Interactive exploration of large-scale images and image data for quantitative tissue cytometry	Winfree et al., 2017b. *J Am Soc Nephrol*. 28:2108-2118 Winfree et al., 2017a.*Transl Res*. 189:1-12	https://github.com/icbm-iupui/volumetric-tissue-exploration-analysis
DeepSynth	Segmentation of nuclei in three-dimensional microscopy images	Dunn et al., 2019. *Sci Rep*. 9:18295 Ho et al., 2017. *IEEE Conference on Computer Vision and Pattern Recognition Workshops (CVPRW)*:834-842 Fu et al., 2017. *IEEE 14th International Symposium on Biomedical Imaging (ISBI 2017)*:704-708	ace@ecn.purdue.edu

## Interactive Visualization of Three-Dimensional Image Volumes – Voxx Software

**FIGURE 1:**
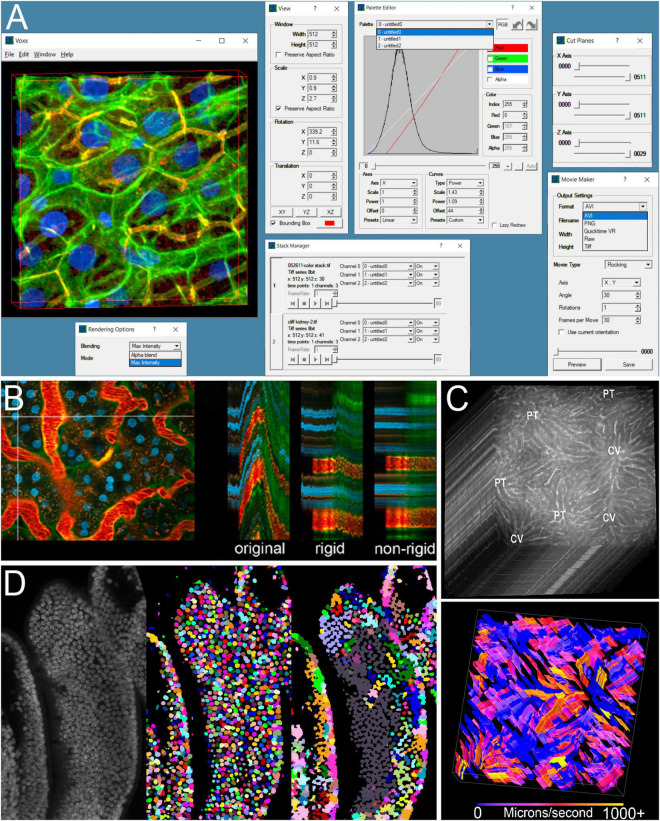
Examples of image processing software developed by the Indiana O’Brien Center. **(A)** Screenshot of Voxx volume visualization software showing the rendered volume, and interactive windows for selecting rendering method, adjusting view and channel palette settings, selecting between multiple volumes, limiting the volume to be displayed, and setting parameters for video outputs. **(B)** Example of IMART image registration. Left – first of a series of images collected over time from the kidney of a living rat. Vertical line indicates region used to generate YT images (two-dimensional images that show the image of a single line, oriented vertically over time, and oriented horizontally). Right – YT images from the original time series, after rigid registration and after rigid and non-rigid registration. **(C)** Example of STAFF microvascular velocity measurements. Top – Series of images collected at the rate of 97.5 frames per second from the liver of a living rat following injection of a fluorescent dextran. Bottom – map of velocities measured over time in which time is presented as a third dimension. **(D)** Comparison of nuclear segmentation results obtained from a 3D volume of mouse intestine (left), using DeepSynth (middle) or CellProfiler (right). Images shown in panels **(B–D)** are modified from previous publications (Dunn et al., 2014, 2019; Clendenon et al., 2019b) and used with permission.

The optical sectioning provided by confocal, and later multiphoton and lightsheet microscopy opened the door to three-dimensional (3D) microscopy. However, when the Indiana O’Brien Center collected its first multiphoton excitation fluorescence image volumes in 2001 visualizing these volumes was challenging. Commonly available software provided static anaglyphs, or sequences of projections, but interactive visualization was limited to scrolling through sequential planes. “Real-time volume rendering” was then an expensive option, requiring costly workstations and surprisingly costly software. However, the rapid growth of video gaming profoundly changed the landscape of computer technology development, moving volume-rendering from a niche scientific market to an enormous consumer market. Jeff Clendenon, a computer engineer in the Indiana O’Brien Center recognized that the graphics capabilities that were once found only on expensive workstations had been reproduced in affordable graphics processors found in personal computers. He proceeded to develop the ground-breaking Voxx scientific volume rendering software, which put real-time volume rendering into the hands of nearly anyone with a personal computer (Clendenon et al., 2002). Voxx (Figure 1A) provides 3D renderings of an image volume (maximum projection or alpha-blending), that update in real-time as the user moves the volume around using a mouse, essentially reproducing the experience of rotating an actual 3D object in space. The ability to interactively manipulate the volume is critical to fully exploring a complex image volume. Voxx also supports the ability to export individual images, or to save a volume rendering sequence as a video for presentations. Over the years since Voxx was released, a variety of volume visualization tools have been developed, both free (e.g., ImageJ) and commercial (e.g., Imaris and Amira). However, because of its unique flexibility and capability, Voxx remains a compelling choice, particularly among free software solutions. Voxx continues to be a mainstay tool of the Indiana O’Brien Center and, as of the time of writing, has been cited in over 100 papers and downloaded more than 6000 times^1^.

## Correcting Motion Artifacts in Intravital Microscopy – Image Motion Artifact Reduction Tool Software

Intravital microscopy has been a core technology of the Indiana O’Brien Center since its inception, and a long-standing goal of the Center has been to promote and facilitate intravital microscopy as a powerful tool for understanding the function of the kidney in health and disease. In our first forays into intravital microscopy we immediately discovered that tissue motion, derived primarily from respiration, represented a significant challenge to high resolution *in vivo* imaging. Subsequent studies of liver, pancreas, lymph nodes, and lung demonstrated that tissue motion was a general problem for intravital microscopy of visceral organs. In contrast to the brain, which can be effectively immobilized using stereotaxic devices attached to the skull, visceral organs move relatively freely in the living animal so that imaging at sub-cellular resolution depends upon methods immobilizing tissue to micron precision. We have since developed robust and reproducible methods for mounting the kidney and other internal organs of rat and mice on the stage of an inverted microscope stage in a way that immobilizes the organ without compromising function (Dunn et al., 2018). Even so, there are occasions when tissue motion cannot be controlled, resulting in studies that cannot be quantified or occasionally, even interpreted.

The problem of tissue motion can be addressed at capture, by gating image collection to avoid respiratory motion [see review in Soulet et al. (2020)], an approach that can be augmented for three-dimensional images, by digital reconstruction (Vladymyrov et al., 2020). For time series studies corrupted by relatively few distorted images, the problem of motion artifacts can be addressed by simply discarding distorted images, or portions of images (Soulet et al., 2013). To address the problem of pervasive image distortion in time series and 3D intravital microscopy, the students from the laboratory of Edward Delp, a Purdue University investigator of the Digital Image Analysis Core of the Indiana O’Brien Center developed novel software to retrospectively correct intravital microcopy images that were compromised by motion artifacts. Based upon an algorithm that seeks to minimize the differences between images, the Image Motion Artifact Reduction Tool (IMART) software can be used to correct motion artifacts in sequences of images collected over time or in three dimensions (Lorenz et al., 2012; Dunn et al., 2014). Unlike other image registration solutions, IMART can be used to correct for both linear (rigid) translations occurring between sequential frames as well as non-linear (warping) distortions occurring within each frame, distortions that are unique to intravital microscopy (Figure 1B). IMART software, which has been downloaded by more than 100 laboratories, has been used to remove motion artifacts from intravital microscopy images collected from the rodent lung and kidney (Presson et al., 2011; Hato et al., 2017), enabling quantitative analysis that would have otherwise been impossible.

## Continuous Measurement of Microvascular Flow Across Entire Optical Sections – Staff Software

Historically, one of the most common applications of intravital microscopy has been the measurement of microvascular flow. The procedure typically involves measuring the displacement of cells or particles in a series of images collected over time from a capillary segment. Cells can be identified either by fluorescent labeling or as shadows in the lumen of the capillary labeled with a fluorescent fluid probe. The velocity of the cells or particles can then be measured either by manually tracking individual cells or by measuring angles in time-distance kymographs. In either case, the process is laborious enough that velocities are typically measured for only a few vascular segments and only for a very brief interval of time. While accurate measurements can be generated in this way, they are susceptible to the spatial and temporal variability that is characteristic of microvascular flow.

To address this problem, the Indiana O’Brien Center worked Sherry Clendenon of the Indiana Biocomplexity Institute to develop an approach for continuous measurement of microvascular flow across entire microscope fields. Using time series images collected by high-speed intravital microscopy, STAFF (Spatial Temporal Analysis of Fieldwise Flow) automatically generates kymographs for each vascular segment in the field, which are then used to generate a complete map of microvascular velocity in each segment across the entire field (Clendenon et al., 2019b; Figure 1C). This approach gives STAFF the unique ability to measure microvascular velocities across entire fields at a temporal resolution on the scale of seconds. Analyses of images collected from the livers of mice demonstrated surprising variability in microvascular flow, with striking differences in flow rates between adjacent sinusoids, and numerous occasions when flow would suddenly stop and later restart. To encourage wide-spread use, STAFF was developed as a freely available plugin to ImageJ and its use is thoroughly described in a JOVE video (Clendenon et al., 2019a).

## Image and Data Exploration for Large Scale Tissue Cytometry – Volumetric Tool for Exploration and Analysis Software

The development of automated microscope systems has enabled researchers to image the distribution of multiple probes at subcellular resolution in centimeter-scale tissue samples. These large and complex image volumes have spurred the development of “tissue cytometry,” an image analysis technique capable of providing complete characterizations of the distribution, interactions and physiology of every cell in an organ (Gerner et al., 2012; Coutu et al., 2017; Halse et al., 2018). However, tissue cytometry represents a relatively new domain of image analysis so that quantitative analysis has largely been accomplished using a combination of custom and/or expensive image analysis software.

To address the need for an accessible solution to tissue cytometry, Seth Winfree, a member of the Indiana O’Brien Center developed VTEA (Volumetric Tool for Exploration and Analysis) (Winfree et al., 2017a,b), a unique software tool that provides a complete integrated workflow supporting every step in tissue cytometry, from segmentation, through classification and quantitation to data analysis via a simple, interactive user interface. A fundamental strength of VTEA is that, by integrating image and data analysis into a single software platform, VTEA expedites and encourages the process of discovery, an exciting aspect of large-scale tissue cytometry. Whereas most imaging studies are predicated on tests of hypotheses, tissue cytometry is typically conducted on images whose size and complexity is such that they contain enormous amounts of additional, latent information that may be apparent only upon exploration. VTEA provides a seamless pipeline between image and data analysis so that the user can, for example, quickly identify specific cell populations in the data space, using either supervised or unsupervised strategies, and visualize their distributions and relations to other cells in the image space. Conversely, the user can also identify interesting regions or cell populations in an image and explore the nature of the cells in these regions in the data space, using either scatterplots or tSNE plots.

VTEA has become a critical tool in the quantitative analysis of tissues by members of the Indiana O’Brien Center and beyond, unlocking the promise of tissue cytometry as tool for biomedical research and discovery. There are several powerful software tools currently available to support tissue cytometry, for example, the Cytomapper and Histocat software developed by the Bodenmiller laboratory (Schapiro et al., 2017; Eling et al., 2020), the Xit software developed by the Schroeder lab (Coutu et al., 2018) and the CytoMAP software developed by the Gerner lab (Stoltzfus et al., 2020). However, none incorporate the entire workflow of image processing, quantitation, visualization, and data analysis into a single continuous bidirectional platform that so effectively encourages exploration and analysis refinement. VTEA-based tissue cytometry has made critical contributions to studies of the processes underlying kidney stone formation conducted as part of a NIH-funded program project (Makki et al., 2020; Winfree et al., 2020) and represents a cornerstone technology of the Indiana University contributions to the Kidney Precision Medicine Project (Winfree et al., 2017a,^2018^; El-Achkar et al., 2021; Ferkowicz et al., 2021). Developed as a plug-in to ImageJ, VTEA is freely available online.

## Online Image Visualization and Analysis – Distributed and Networked Analysis of Volumetric Image Data High Performance Image Analysis System

Capable of defining the distribution of multiple molecular species at subcellular resolution over regions spanning the full extent of the cortex and medulla, large-scale microscopy image volumes are enormously rich in potential information. However, extracting this information is challenging, not only because of the unique challenges of 3D image analysis, but also because the size and complexity of these image volumes are incompatible with resources available to most researchers. Large-scale image data places an enormous burden on computer and network infrastructure. A four- channel image volume, collected at subcellular resolution to a depth of 100 microns from a 5 × 6 mm region requires nearly 200 gigabytes of digital storage space. A complete study, which might involve comparison of multiple conditions, each with a reasonable number of replicates, could thus easily involve tens of terabytes of data. Managing data of this magnitude requires extensive and sophisticated computer hardware and network infrastructure beyond that available at most institutions. And the challenges of visualizing and quantifying 3D images, discussed previously, become much larger in image volumes of this scale.

To encourage the application of large-scale tissue cytometry by a broader range of investigators, the Indiana O’Brien Center has a developed an approach to large-scale microscopy that both removes most of these challenges. The O’Brien Center 3D Tissue Imaging Core provides a service whereby samples sent to the Center are imaged using one of the confocal or multiphoton microscopes of the Indiana Center for Biological Microscopy, and the resulting data archived at Indiana University, thus eliminating an investigator’s need for extensive storage and network capabilities. The resulting images are also uploaded to a powerful online server system, the DINAVID (Distributed and Networked Analysis of Volumetric Image Data) high performance image analysis system. Hosted by Indiana University and developed by the laboratories of Edward Delp at Purdue and Paul Salama at IUPUI, the DINAVID system is designed to provide remote users throughout the world with an intuitive interface to their image data, supporting interactive visualization, quantitative analysis, and exploration. The DINAVID system is continuously updated with new tools as they are developed by the O’Brien Center Digital Image Analysis Core, including novel methods of 3D segmentation, as described below.

## Nuclear Segmentation Using a Convolutional Neural Network Trained in Synthetic Data – Deepsynth Segmentation Software

Tissue cytometry is formally similar to flow cytometry, except that whereas in flow cytometry the sample is passed through a detector, in tissue cytometry, the detector is passed over the sample. However, unlike flow cytometry, where individual cells are physically separated from one another for quantification, tissue cytometry is complicated by the need for image analysis techniques to distinguish individual cells that are packed into a tissue. The process of distinguishing individual cells, cell “segmentation” is the critical first step in tissue cytometry. In the absence of membrane markers to delineate cell boundaries, individual cells are typically distinguished by their nuclei. Cells are then classified into specific cell types based upon the presence of specific markers in the regions surrounding each nucleus.

Numerous approaches have been developed to segment nuclei in two-dimensional images, supporting automated analysis of thin tissue sections and cells grown in culture. Historically, these approaches have been based upon traditional, morphological image processing operations but increasingly, investigators are demonstrating that deep-learning techniques frequently provide results that are more accurate (Caicedo et al., 2019a,b). Moreover, unlike morphological techniques that typically need to be tuned to the specific characteristics of each image, deep-learning techniques generally provide results that are robust across different images. However, that robustness is typically obtained only when the network is provided with a large amount high-quality “training” data – a library of images that have been manually annotated that are used by the network to “learn” the qualities of nuclei. As manual annotation is a laborious process, training is typically the rate-limiting step in the application of deep-learning techniques to segmentation. The barrier of manual annotation has been addressed in various ways, including side-stepping the annotation process and training networks using publicly available annotated datasets (Caicedo et al., 2019a; Stringer et al., 2021), using crowd-sourcing to annotate images (Moen et al., 2019), or using transfer learning, a process in which a network trained on a large amount of data is refined using a much smaller dataset (Zaki et al., 2020). Interested readers are directed to a recently published review of open-source, deep-learning software for segmentation of biological images (Lucas et al., 2021).

Segmentation of nuclei in three-dimensional tissues is significantly more challenging, in part because of the relatively poor axial resolution of optical microscopy. Accordingly, techniques for segmentation of nuclei in three-dimensional tissues are much less developed, seriously limiting biologists’ ability to quantitatively analyze three-dimensional image volumes. As with two-dimensional segmentation, deep-learning represents an exciting approach to segmentation of three-dimensional images. However, the already tedious task of generating training data is even more onerous in three dimensions. As with two-dimensional data, network performance depends upon annotation of hundreds, if not thousands of nuclei. Extending segmentation to three dimensions means that each nucleus must be manually annotated in multiple focal planes, including those collected from the top and bottom boundaries that are especially difficult to reproducibly delineate.

The Digital Image Analysis Core of the Indiana O’Brien Center addressed this problem by developing DeepSynth, a convolutional neural network trained on synthetic images, essentially eliminating the tedious task of manual annotation (Fu et al., 2017; Ho et al., 2017, 2018; Dunn et al., 2019). As compared with 3D segmentation software based upon traditional morphological segmentation techniques, DeepSynth provides segmentations that are more accurate, particularly for challenging image volumes (Figure 1D). A second benefit of the DeepSynth approach is that the quality of segmentations are more consistent throughout large image volumes. At the time of writing, the DeepSynth software, which is freely available from the O’Brien website, has been downloaded by 18 laboratories.

## Future Directions of the Digital Image Analysis Core of the Indiana O’Brien Center

The Digital Image Analysis Core is dedicated to the development of image analysis software to further the research of renal investigators. An overarching theme of the core is that the images of biological microscopy are rich in information, and extracting that information depends upon hands-on exploration and analysis by biologists who are not necessarily experts in digital image analysis. Thus image analysis software should be accessible, interactive, and user-friendly. Going forward, the core will continue to improve and refine deep learning-based methods of image segmentation by implementing more accurate models of synthetic data, e.g., by incorporating models of objective lens point-spread functions. The core is also working on methods to address the spatial variability in segmentation quality that we observe in large, three-dimensional image volumes. Insofar as segmentation is fundamental to image quantification, spatial variability in segmentation quantity directly impacts the reliability of tissue cytometry. Since it is impractical to measure segmentation quality at every point in a large image volume, methods are needed to estimate segmentation quality and to convert these estimates into confidence maps that can be used to inform interpretation of cytometry measurements. Finally, the core is also exploring how deep learning can be expanded into additional aspects of image analysis in tissue cytometry, including noise reduction, spectral deconvolution and cell classification.

## Author Contributions

The author confirms being the sole contributor of this review and has approved it for publication.

## Conflict of Interest

The author declares that the research was conducted in the absence of any commercial or financial relationships that could be construed as a potential conflict of interest.

## Publisher’s Note

All claims expressed in this article are solely those of the authors and do not necessarily represent those of their affiliated organizations, or those of the publisher, the editors and the reviewers. Any product that may be evaluated in this article, or claim that may be made by its manufacturer, is not guaranteed or endorsed by the publisher.

## Footnotes

1
http://web.medicine.iupui.edu/ICBM/software
